# Interactions of the Human MCM-BP Protein with MCM Complex Components and Dbf4

**DOI:** 10.1371/journal.pone.0035931

**Published:** 2012-04-23

**Authors:** Tin Nguyen, Madhav Jagannathan, Kathy Shire, Lori Frappier

**Affiliations:** Department of Molecular Genetics, University of Toronto, Toronto, Canada; St. Georges University of London, United Kingdom

## Abstract

MCM-BP was discovered as a protein that co-purified from human cells with MCM proteins 3 through 7; results which were recapitulated in frogs, yeast and plants. Evidence in all of these organisms supports an important role for MCM-BP in DNA replication, including contributions to MCM complex unloading. However the mechanisms by which MCM-BP functions and associates with MCM complexes are not well understood. Here we show that human MCM-BP is capable of interacting with individual MCM proteins 2 through 7 when co-expressed in insect cells and can greatly increase the recovery of some recombinant MCM proteins. Glycerol gradient sedimentation analysis indicated that MCM-BP interacts most strongly with MCM4 and MCM7. Similar gradient analyses of human cell lysates showed that only a small amount of MCM-BP overlapped with the migration of MCM complexes and that MCM complexes were disrupted by exogenous MCM-BP. In addition, large complexes containing MCM-BP and MCM proteins were detected at mid to late S phase, suggesting that the formation of specific MCM-BP complexes is cell cycle regulated. We also identified an interaction between MCM-BP and the Dbf4 regulatory component of the DDK kinase in both yeast 2-hybrid and insect cell co-expression assays, and this interaction was verified by co-immunoprecipitation of endogenous proteins from human cells. *In vitro* kinase assays showed that MCM-BP was not a substrate for DDK but could inhibit DDK phosphorylation of MCM4,6,7 within MCM4,6,7 or MCM2-7 complexes, with little effect on DDK phosphorylation of MCM2. Since DDK is known to activate DNA replication through phosphorylation of these MCM proteins, our results suggest that MCM-BP may affect DNA replication in part by regulating MCM phosphorylation by DDK.

## Introduction

The initiation of DNA replication in all eukaryotes involves the assembly of pre-replicative complexes (pre-RC) at the origins of replication in the G1 phase of the cell cycle, followed by the activation of the pre-RC at the onset of S phase. A critical component of the pre-RC is the minichromosome maintenance (MCM) complex, which is comprised of MCM proteins 2 through 7 that form a hexameric ring [1]. Double hexamers of the MCM complex are loaded onto each origin by the action of Cdc6 and Cdt1 and, upon entry into S phase, this complex is activated by phosphorylation by the S-phase specific kinase DDK (Dbf4/Drf1-dependent kinase) (reviewed in [2], [3]). DDK is comprised of the cdc7 kinase and either Dbf4 or Drf1 regulatory subunits. DDK phosphorylates MCM2, MCM4 and MCM6 and triggers the recruitment of Cdc45 and GINS to form the Cdc45, MCM, GINS (CMG) complex that functions as the replicative helicase [4], [5], [6], [7], [8], [9], [10].

MCM2-7 are members of the AAA+ ATPase family of proteins and can hydrolyse ATP when combined into particular MCM dimer pairs or larger complexes [1], [11], [12], [13]. MCM4,6,7 and MCM4,7 can form stable hexamers that have DNA helicase activity [13], [14], [15], although MCM2-7 hexamers are thought to be the active form of the helicase in the context of the CMG complex [4], [11]. MCM proteins are present in cells at levels considerably above what is needed to unwind DNA at replication forks, suggesting that they can play additional roles [1]. Indeed, excess chromatin-associated MCM complexes have been found to be important under conditions of replication stress, where they activate dormant origins to ensure that DNA replication continues when replication forks stall [16], [17]. In addition, some MCM subunits appear to have additional functions that are independent from DNA replication [18], [19], [20].

In efforts to more completely understand the functions and regulation of MCM proteins, human MCM6 and MCM7 subunits were subjected to *in vivo* tandem affinity purification (TAP) tagging, revealing that these proteins not only interact with the other MCM proteins, but also co-purify with a previously unstudied protein that we named MCM-BP [21]. MCM-BP is conserved in most eukaryotes (except budding yeast and *C. elegans*) and has only limited homology to MCM proteins. TAP-tagging or immunoprecipitation of MCM-BP from human cells, Xenopus egg extracts and *Schizosaccharomyces pombe* recovered MCM 3 through 7 but not MCM2 [21], [22], [23], [24]. Conversely TAP-tagging of MCM2 in human cells recovered MCM3 through 7 but not MCM-BP, suggesting that alternative MCM complexes exist that contain either MCM-BP or MCM2. However, MCM-BP can also interact with some MCM proteins individually as interactions between *Xenopus* MCM-BP and MCM7 and between *Arabidopsis thaliana* MCM-BP (ETG1) and MCM5 have been reported [22], [25]. Human MCM-BP was shown to interact with the MCM4,6,7 subcomplex but did not inhibit the *in vitro* helicase activity of this complex [21].

Like MCM proteins, MCM-BP is a nuclear protein found in both chromatin-associated and soluble forms. In human cells, a proportion of MCM-BP is chromatin associated through G1 and S, with preferential origin localization at G1/S, then dissociates from the DNA at late G2 or early M, resembling the pattern of chromatin association of the MCM complex [21]. In *S. pombe*, MCM-BP is encoded by an essential gene (*mcb1*) [23]. Deletion of *pombe mcb1* resulted in gradual cell cycle arrest and a cdc phenotype, whereas Mcb1 overexpression induced DNA damage and Chk1 activation [23]. Similarly Mcb1 inactivation in *S. pombe* or MCM-BP depletion in human cells resulted in increased DNA damage and G2 checkpoint activation [24], [26]. In human cells, MCM-BP depletion also leads to centrosome amplification and abnormal nuclear morphology, which may be due to the G2 checkpoint activation [26]. The MCM-BP homologue in *A. thaliana* (ETG1) was identified as an E2F target and loss of ETG1 was found to reduce DNA replication, activate the G2 checkpoint and reduce sister chromosome cohesion [25], [27]. All of these observations point to an important role of MCM-BP in DNA replication.

One of the roles of MCM-BP appears to be in unloading the MCM complex from chromatin after DNA synthesis as suggested by studies in *Xenopus* egg extracts, where depletion of MCM-BP from the extracts reduced the dissociation of MCMs from the chromatin at the end of S phase [22]. However, MCM-BP depletion in human cells not only increased the levels of chromatin-associated MCMs at the end of S phase, but resulted in a similar increase in the soluble levels of MCMs throughout S phase [22], [26], suggesting that MCM-BP makes multiple contributions to DNA replication. A major difference between human cells and the *Xenopus* extract system is that MCM-BP only enters the Xenopus nuclei in mid S phase, whereas MCM-BP in human cells is largely nuclear throughout the cell cycle, where it may affect other functions of the MCM complex proteins.

While TAP-tagging and endogenous immunoprecipitations performed in human cells suggest that MCM-BP can form a hexameric complex with MCM3-7, it is not clear how MCM-BP interacts with this complex nor has it been determined whether human MCM-BP can interact with any individual MCM subunits. In addition, while recruitment of DDK to the MCM2-7 complex is known to be important for DNA replication, it is not known whether MCM-BP or MCM-BP-containing MCM complexes associate with this kinase. Here we examine the associations of MCM-BP with individual MCM complex proteins and DDK. We show that MCM-BP is capable of interacting with any individual MCM protein but interacts most strongly with MCM4 and MCM7. In addition, we show that MCMBP and MCM proteins form part of a large complex that forms at mid to late S phase. Finally, we identify an interaction between MCM-BP and the Dbf4 subunit of DDK and show that MCM-BP can decrease MCM phosphorylation by DDK *in vitro*.

## Results

### MCM-BP Can Interact with any Individual MCM Subunit

**Figure 1:**
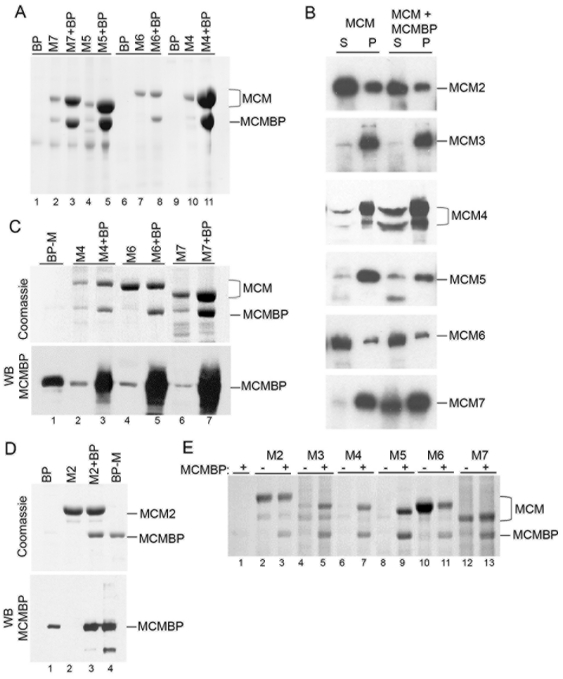
MCM-BP interacts with individual MCM proteins. A. Affinity tagged MCM4, 5, 6 or 7 (M4, M5, M6, M7) were expressed from baculoviruses in the presence (+BP) or absence of a baculovirus expressing MCM-BP. Cell lysates were then incubated with the appropriate affinity resin and bound protein were eluted by boiling in SDS buffer. Cells infected only with the MCM-BP baculovirus (BP lanes) were included as a negative control for each resin. A Coomassie stained gel of the eluted proteins is shown. B. The indicated MCM protein was expressed in insect cells with or without MCM-BP. Cell lysates were clarified by centrifugation and equal fractions of the soluble (S) and pelleted proteins (P) were analysed by SDS-PAGE and Coomassie staining. C. Affinity tagged MCM4, 6 or 7 were expressed with or without MCM-BP and recovered on affinity resin as in A. The top panel shows the Coomassie stained gel of the eluted proteins. The same samples were also immunoblotted using anti-MCM-BP antiserum (bottom panel). Purified MCM-BP (200 ng) was loaded as a marker in lane 1 (BP-M). D. Strep-tagged MCM2 was expressed in insect cells with or without MCM-BP, recovered on Strep-T actin resin and processed as in C. MCM-BP expressed on its own was also incubated with Strep-T actin resin to establish the level of nonspecific interaction with the resin (lane 1). Purified MCM-BP (1 µg) was loaded as a marker in lane 4 (BP-M). E. The indicated 6-His-tagged MCM protein was expressed with or without MCM-BP (as indicated) and recovered on Ni-NTA resin. In lane 1, MCM-BP was expressed on its own to determine the level of nonspecific binding to the Ni-NTA resin. A Coomassie stained gel of the eluted proteins is shown.

We examined the ability of MCM-BP to interact with MCM proteins by co-expressing MCM-BP with individual MCM proteins in insect cells. To this end, insect cells were infected with a baculovirus expressing affinity tagged MCM4, MCM5, MCM6 or MCM7 (Strep-MCM4, FLAG-MCM5, HA-MCM6, FLAG-MCM7) with or without a second baculovirus expressing untagged MCM-BP. Tagged MCM proteins were then recovered on the appropriate affinity resin (Strep T actin for MCM4, anti-HA for MCM6 and anti-FLAG for MCM5 and 7) and recovery of the MCM protein and MCM-BP was assessed by Coomassie staining (Figure 1A). Each MCM protein recovered MCM-BP, whereas MCM-BP was not recovered on the affinity resin in the absence of the MCM protein. In each case, MCM-BP recovery was proportional to the amount of MCM protein recovered suggesting a stoichiometric interaction. We also found that the presence of MCM-BP dramatically increased the recovery of MCMs 4, 5 and 7, also supporting an interaction of MCM-BP with these proteins. The increased recovery of these MCM proteins in the presence of MCM-BP may be due to the increased solubility of MCMs 4, 5 and 7 when expressed in the presence of MCM-BP. As shown in Figure 1B, these MCM protein are largely insoluble when expressed in insect cells on their own, but the amount of MCM4, 5 or 7 in the soluble fraction is increased when co-expressed with MCM-BP (most obviously for MCM7). On the other hand, MCM6 is largely soluble on its own which may be why its recovery is not increased by MCM-BP, even though these proteins can interact.

In the experiments in Figure 1A, we noticed that the MCM proteins expressed in the absence of human MCM-BP, often recovered a small amount of protein that ran at a size consistent with MCM-BP, suggesting that it might be the endogenous insect MCM-BP. To investigate this further, we repeated the affinity purification experiments with MCM 4, 6 and 7 in the presence and absence of MCM-BP and performed Western blots on the recovered proteins with MCM-BP antibody (Figure 1C). All three of these MCM proteins recovered a band the size of MCM-BP in the absence of the MCM-BP baculovirus, and the Western blot confirmed that this band was likely endogenous insect MCM-BP since it was recognized by anti-MCM-BP antibody. This suggests that interactions of these MCM proteins with MCM-BP do not require high levels of MCM-BP and can occur with the endogenous insect MCM-BP.

MCM2 is the only MCM complex protein that is not recovered with MCM-BP isolated from human cells. This could either mean that MCM-BP binds the MCM3-7 complex or that MCM-BP forms dimer pairs with MCM3 through 7 and is unable to dimerize with MCM2. To differentiate between these possibilities we tested whether MCM-BP could bind MCM2 in pulldown assays in insect cells. When Strep-tagged MCM2 was co-expressed in insect cells with untagged MCM-BP, MCM-BP was recovered with MCM2 on the Strep-T actin resin, but much less MCM-BP was recovered on the resin in the absence of MCM2 (not detectable by Coomassie staining), indicating that the two proteins can interact (Figure 1D; compare lanes 1 and 3). However, unlike the interaction with MCM4, 5 and 7, the presence of MCM-BP did not increase the recovery (Figure 1D; compare lanes 2 and 3) or solubility (Figure 1B) of MCM2. Also MCM2 did not recover detectable amounts of endogenous insect MCM-BP (Figure 1D, lane 2), suggesting that the MCM2-MCM-BP interaction requires higher levels of proteins and hence is a lower affinity interaction than MCM-BP interactions with the other MCM proteins.

Finally, we generated baculoviruses expressing each MCM protein with a 6-His-tag and expressed these proteins with and without MCM-BP (with a Strep tag but lacking a His-tag) in insect cells in order to compare MCM recovery using the same affinity resin (Figure 1E). The results confirmed that all MCM proteins interact to some degree with MCM-BP and that the presence of MCM-BP increases the recovery of all MCMs except MCM2 and MCM6.

### MCM-BP Interacts Most Stably with MCM4 and MCM7

**Figure 2:**
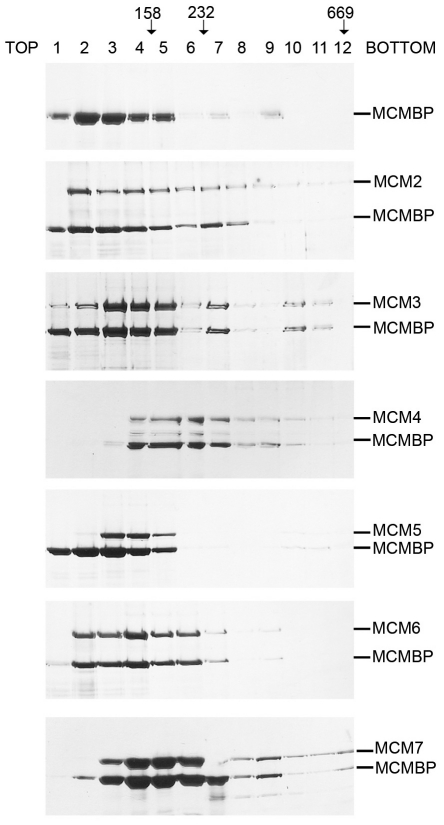
Glycerol gradient sedimentation analyses of MCM-MCM-BP pairs. Affinity tagged MCM proteins were co-expressed with nontagged MCM-BP in insect cells then recovered on and eluted from the appropriate affinity resin as described in Materials and Methods. His-tagged MCM-BP was also expressed on its own and recovered on Ni-NTA resin (top panel). 150–200 µg of protein was then subjected to centrifugation through a glycerol gradient for 16 hours. 24 500 µl fractions were collected from the top of the gradient and 35 µl samples of each were analysed by SDS-PAGE and silver staining. Only the top 12 fractions are shown since they contained all of the protein. The peak positions of molecular weight markers aldolase (158 kDa), catalase (232 kDa) and thyroglobulin (669 kDa) are indicated at the top of the gels.

The pull-down assays performed above can detect relatively weak or transient protein interactions. To get a better sense of the strength of the MCM-BP interaction with individual MCM proteins, MCM-BP-MCM protein pairs were expressed in insect cells and isolated by virtue of the affinity tag on the MCM protein or a His-tag on MCM-BP (for MCM3 interaction only where MCM3 has no tag). The recovered proteins were then subjected to glycerol gradient sedimentation, which only detects protein interactions that are stable enough to remain intact during a 16 hour spin through the glycerol gradient. MCM-BP on its own migrates at the top of the gradient, peaking at fractions 2 to 3 (Figure 2, top panel), and a stable interaction with any MCM protein would be expected to shift this peak towards the bottom of the gradient due to its larger size. When the MCM-BP-MCM pairs were analysed on identical gradients, only MCM4 and MCM7 pairs caused a significant shift in the migration of the MCM-BP peak, such that the bulk of the MCM-BP migrated in fractions 4 to 6 and corresponded closely to the migration of MCM4 and MCM7. This indicates that MCM-BP formed a stable complex with MCM4 and MCM7. Other MCM proteins caused the broadening of the MCM-BP peak towards the bottom of the gradient to various degrees with a significant proportion of MCM-BP remaining at fractions 2–3. This is suggestive of interactions that were dissociating during the course of the gradient. Therefore this assay indicates that MCM-BP interacts most strongly with MCM4 and MCM7.

### Effects of Salt and Detergent on MCM-BP-MCM Interactions

**Figure 3:**
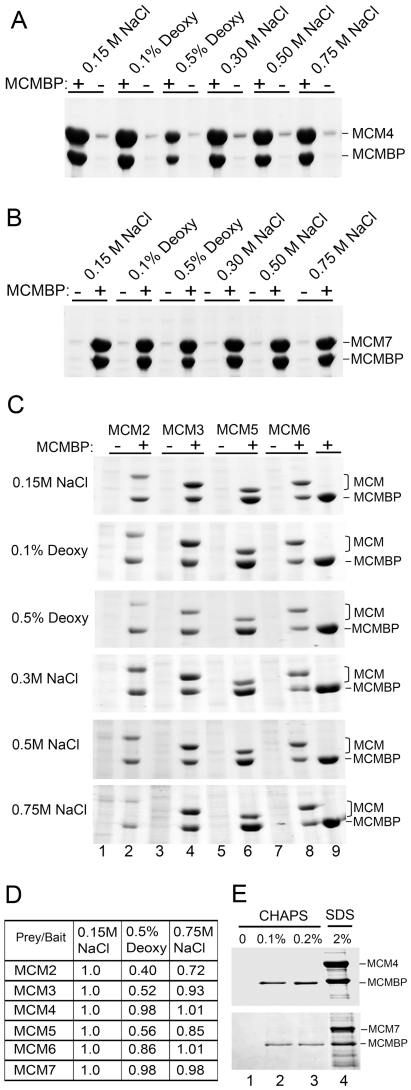
Effect of salt and detergent on MCM-BP interactions with MCM proteins. A and B. Strep-tagged MCM4 (A) or FLAG-tagged MCM7 (B) were expressed in insect cells with or without untagged MCM-BP then recovered from cell lysates on Strep-T actin (A) or anti-FLAG (B) resin. Coomassie stained gels are shown. The salt and deoxycholate (Deoxy) concentrations in the lysis buffer are indicated. Samples containing 0.1% and 0.5% deoxycholate also contain 150 mM NaCl. C. Untagged MCM2, MCM3, MCM5, or MCM6 were expressed in insect cells with and without His-tagged MCM-BP then cells were lysed in the same buffers as in A and B as indicated. Cell lysates were then incubated with nickel resin and bound proteins eluted with 250 mM imidazole. Lane 9 is a negative control in which His-MCM-BP was expressed on its own. D. The ratio of the recovered proteins (untagged∶tagged protein) in A–C was determined using ImageQuant quantification of the protein bands and is shown relative to the ratio seen for the 0.15 M NaCl condition (set to 1). E. His-tagged MCM4 or MCM7 was expressed with untagged MCM-BP in insect cells. Cells were lysed in lysis buffer containing 150 mM NaCl (no deoxycholate) and lysates were incubated with nickel resin. After washing, the resin was incubated in buffer containing 0, 0.1% or 0.2% CHAPS, or boiled in 2% SDS sample buffer to show the proteins initially bound to the resin.

We further investigated the strength of the interaction of MCM-BP with MCM4 and MCM7, by co-expressing the proteins in insect cells and isolating the MCM protein on affinity resin under various conditions. As shown in Figure 3A and B, MCM-BP was efficiently recovered with MCM4 or MCM7 under a variety of conditions including NaCl concentrations up to 0.75 M and deoxycholate up to 0.5% (with 150 mM NaCl). Therefore these interactions are extremely stable and are particularly resistant to high salt concentrations. We then performed similar interaction assays expressing His-tagged MCM-BP with the other MCM proteins (untagged) from insect cells and isolating MCM-BP on nickel resin under the same conditions as above (Figure 3C). The ratio of the two recovered proteins in the 0.5% deoxycholate and 0.75M NaCl conditions was determined and is shown relative to the ratio in the 0.15 M NaCl condition (set to 1; Figure 3D). MCM-BP interactions with each of the MCM proteins were observed under all of the conditions tested, although the recovery of MCM 2, 3 and 5 with MCM-BP was noticeably decreased in 0.5% deoxycholate and the recovery of MCM2 was also decreased in 0.75M NaCl concentrations. These results are in keeping with the glycerol gradient analyses that MCM-BP interactions with MCM 2, 3 and 5 are less stable than with MCM 4 and 7.

Finally, we also sought conditions that could disrupt the stable interactions of MCM-BP with MCM4 and 7. To this end, His-tagged MCM4 or MCM7 was coexpressed with untagged MCM-BP in insect cells, then cells were lysed and the MCM-MCM-BP complexes were recovered on nickel resin. Incubation of the resin with various buffers showed that MCM-BP was released from MCM4 or MCM7 when small amounts of CHAPS (0.1–0.2%) were included in the wash buffer (Figure 3E), showing the sensitivity of the MCM-BP-MCM interactions to this nondenaturing zwitterionic detergent.

### Analysis of MCM-BP and MCM Complexes in Human Cells

**Figure 4:**
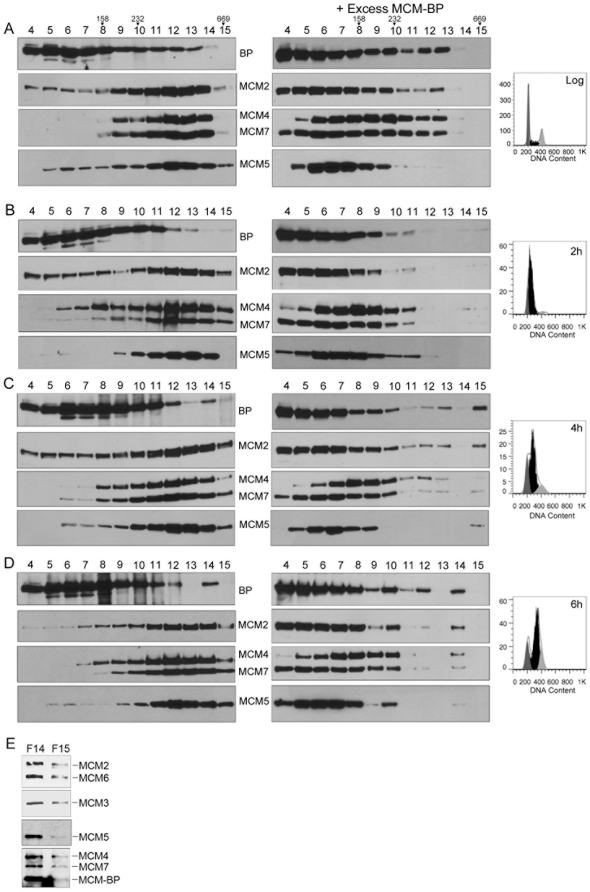
Analysis of MCM and MCM-BP complexes in human cell lysates. A. Cell lysates from log-phase HeLa cells were subjected to glycerol gradient sedimentation and equal volume fractions were collected from the top of the gradient. An equal volume of each fraction was analysed by Western blotting using antibodies against the indicated MCM protein or MCM-BP (BP; left panels). The same samples were also analysed after the addition of purified MCM-BP to the lysate (right panels). Note that the MCM-BP blots on the right are exposed for shorter times than those on the left due to the higher amounts of MCM-BP. The peak positions of molecular weight markers aldolase (158 kDa), catalase (232 kDa) and thyroglobulin (669 kDa) are indicated at the top of the gels (which also apply to B–D). The DNA profile as determined by flow cytometry is shown on the right, where G1 cells are shown in dark grey, S cells are shown in black and G2/M cells are shown in light grey. B–D. HeLa cells blocked at the G1/S boundary with double thymidine treatment were released into S phase for 2 (B), 4 (C) or 6 (D) hours, then cell lysates were prepared and analysed as in A. E. MCM-BP was immunoprecipitated from fractions 14 (F14) and 15 (F15) in D and immunoblots were performed for MCM-BP and each of the MCM proteins.

While previous studies suggest that MCM-BP can form complexes with MCM proteins [21], it is not clear how frequently and under what circumstances these complexes form in cells. We examined the state of MCM-BP complexes in human cells by performing glycerol gradient sedimentation analysis of log-phase HeLa cell extracts and immunoblotting for MCM-BP and MCM proteins (Figure 4A, left panel). We found that the MCM proteins co-migrate in the gradient at a size consistent with a hexameric complex and that a proportion of the MCM-BP also overlapped with this peak, suggesting that some of the MCM complexes may contain MCM-BP. However, the majority of the MCM-BP peaked at a higher position in the gradient suggesting that it was not MCM-associated. Since *Xenopus* MCM-BP has been found to be able to dissociate MCM complexes [22], we also examined whether the addition of excess exogenous MCM-BP to the cell lysate affected the migration of the MCM proteins (Figure 4A, right panel). The addition of MCM-BP resulted in dissociation of the MCM complex such that the MCM proteins now largely migrated at positions indicative of individual proteins or smaller complexes. Note that the right MCM-BP panel is a lighter exposure than the left MCM-BP panels due to the higher levels of MCM-BP present.

Since MCM complexes and MCM-BP function as DNA replication proteins in S phase, similar experiments as above were performed with HeLa cells that were synchronized at the G1/S boundary (using a double thymidine block) and released into S phase. The results at the 2 hour (early S) time point (Figure 4B, left panel) were very similar to those in log-phase cells, except that there may be less MCM-BP at the position of the large MCM complex. At mid to late S-phase (4 and 6 hour time points; Figure 4C and 4D, left panels), a small fraction of the MCM-BP formed a second peak (at fraction 14) that overlapped with the MCM hexamer peak. The addition of excess MCM-BP to the extracts from any of these time points resulted in dissociation of the MCM hexamers, but in the mid to late S extracts, a small amount of the MCMs were still detected in a large complex that also contained MCM-BP, migrating at fractions 13–15 (Figure 4B–D, right panels). The formation of this large MCM-BP-containing complex has been seen in multiple experiments. To further determine if MCM-BP and MCM proteins were present in the same large complex in late S, MCM-BP was immunoprecipitated from fraction 14 in Figure 4D and the recovery of MCM proteins was examined. MCM2 to 7 were all found to co-immunoprecipitate with MCM-BP indicating that they are part of the same complex. For comparison, we performed the same immunoprecipitation from fraction 15, which has little MCM-BP. As expected less MCM-BP immunoprecipitated from this fraction and the recovery of each MCM protein also decreased. The results suggest that, while the majority of the MCM-BP is not normally present in MCM complexes, some of the MCM-BP is associated with large MCM complexes at mid to late S. The data also indicates that human MCM-BP, like *Xenopus* MCM-BP, has the ability to disrupt MCM hexamers.

### MCM-BP Interacts with Dbf4

**Figure 5:**
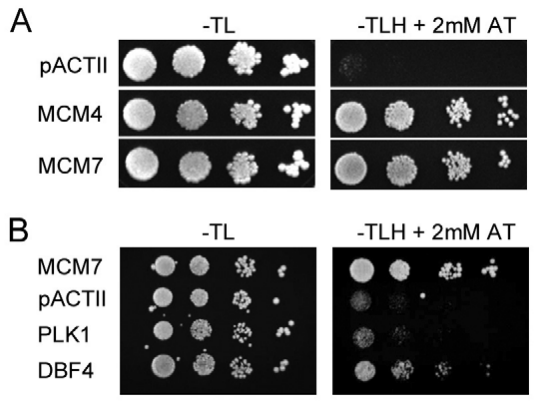
Yeast 2-hybrid assays of MCM-BP interactions. MCM-BP fused to the LexA DNA binding domain was expressed in yeast containing a histidine gene under LexA control, along with the indicated proteins expressed as activation domain fusions from pACTII. The empty pACTII plasmid was also expressed with MCM-BP as a negative control. Ten-fold serial dilutions of the yeast cultures were spotted on plates that select for the two expression plasmids with (right panels) or without (left panels) selection for histidine expression.

Interactions of the MCM2-7 complex with DDK are critical for DNA replication and can be detected by yeast 2-hybrid assays performed with MCM2, 3, 4 and 7 subunits and the Dbf4 regulatory component of the kinase [28], [29]. Since MCM-BP interacts with MCM proteins and can associate with cellular origins at G1/S (when DDK is active), we wanted to determine if MCM-BP could interact with Dbf4. We tested this initially using yeast 2-hybrid assays in which MCM-BP fused to the LexA DNA binding domain must interact with a target protein fused to an activation domain in order to drive expression of a histidine gene under control of LexA binding sites, enabling the yeast to grow on plates lacking histidine. First we showed that MCM-BP could mediate the expected interactions with MCM proteins in the 2-hybrid system, as interactions with MCM4 and MCM7 were readily detected by the vigorous growth of the yeast on plates lacking histidine and containing 2 mM aminotriazole, which was not seen when MCM-BP was expressed with the activation domain alone (Figure 5A). We then used the same system to test MCM-BP binding to Dbf4 and another unrelated kinase, Plk1. As shown in Figure 5B, no interaction of MCM-BP with Plk1 was detected, however MCM-BP was found to bind Dbf4, resulting in growth of the yeast on the plates lacking histidine to an extent slightly less than that seen with MCM-BP and MCM7.

**Figure 6:**
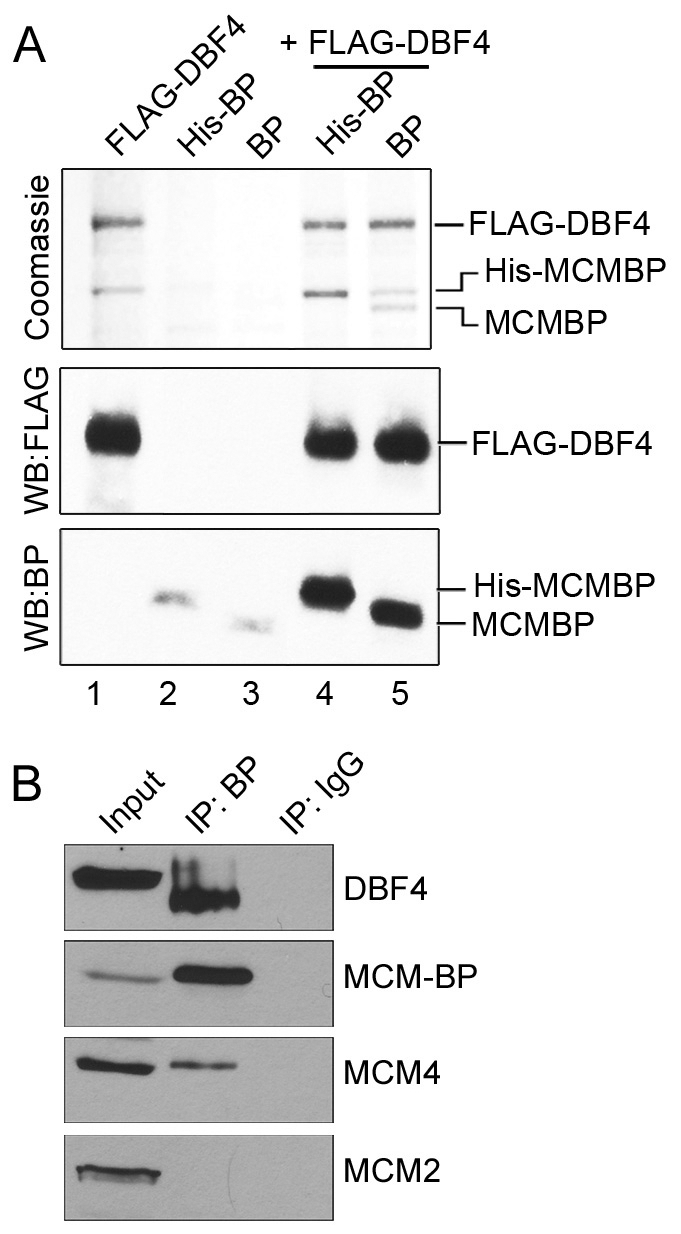
Interactions of MCM-BP with Dbf4. A. Untagged (BP) or His-tagged MCM-BP (His-BP) were expressed in insect cells with or without FLAG-tagged Dbf4 then soluble proteins were recovered on anti-FLAG resin. Eluted proteins are shown by Coomassie staining (top panel) and Western blots for FLAG (middle panel) and MCM-BP (bottom panel). B. Endogenous MCM-BP was immunoprecipitated from HeLa cell lysates (IP:BP) followed by Western blotting for MCM-BP, Dbf4, MCM2 and MCM4. Immunoprecipitation with a negative control IgG is also shown (IP:IgG) as is 5% of the starting lysate (Input). Note that different exposure times were used in each Western blot to maximize protein detection.

We also tested the ability of MCM-BP and Dbf4 to interact when co-expressed in insect cells. To this end, insect cells were infected with a baculovirus expressing untagged MCM-BP or His-tagged MCM-BP with or without a baculovirus expressing FLAG-tagged Dbf4, then proteins were recovered on anti-FLAG resin (Figure 6A). In the presence of Dbf4, both version of MCM-BP were recovered on the resin, as observed by both Coomassie staining (Figure 6A., top panel) and Western blotting for MCM-BP (bottom panel), but very little MCM-BP associated with the resin in the absence of Dbf4 (only detected by Western blotting). Note that a background band the size of His-MCM-BP is also recovered with Dbf4, even when it is expressed in the absence of MCM-BP (seen in lanes 1 and 5, top panel) but this band is not recognized by the MCM-BP antibody. Therefore the results indicate that MCM-BP can interact with Dbf4 in this system.

Finally we asked whether an interaction between endogenous MCM-BP and Dbf4 could be detected in human cells, by immunoprecipitating MCM-BP from HeLa cells and blotting for Dbf4. As shown in Figure 6B, Dbf4 was efficiently recovered with MCM-BP but was not detected when negative control IgG was used. Consistent with our previous findings [21], MCM4 but not MCM2 also co-immunoprecipitated with MCM-BP. The Dbf4 that was recovered with MCM-BP migrated slightly faster than the main Dbf4 band in the lysate and this shift in migration has also been reported for Dbf4 immunoprecipitated with Cdc7 from human cells [30].

### MCM-BP is not a DDK Substrate but Inhibits MCM Phosphorylation by DDK

**Figure 7:**
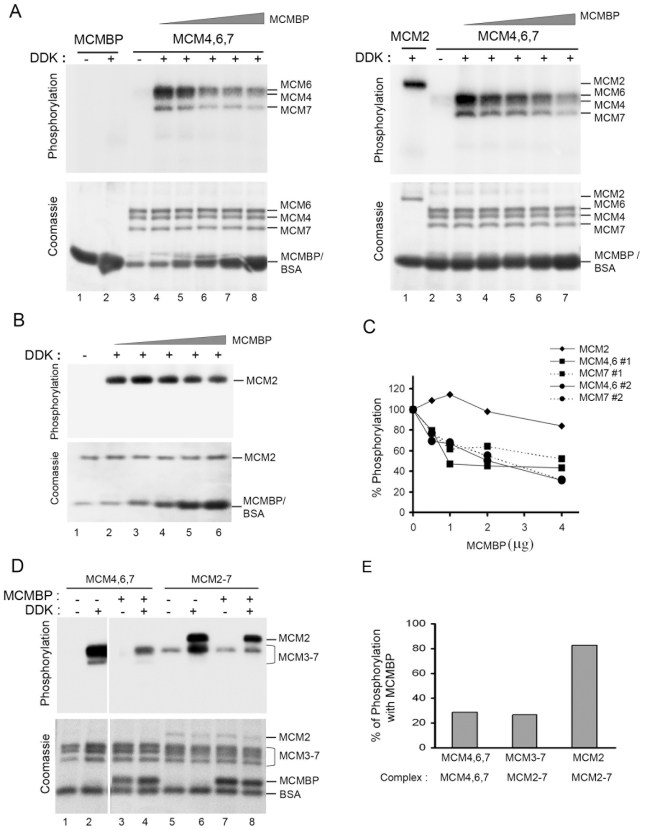
MCM-BP can decrease phosphorylation by DDK but is not a substrate. A. 2 µg of MCM4,6,7 complex was pre-incubated with increasing amount of MCM-BP (0,0.5, 1.0, 2.0 and 4.0 µg; lanes 4–8 in left panel and 3–7 in right panel) then combined with 250 ng of purified Cdc7-Dbf4 in a reaction containing γ-^32^P-ATP. After 30 min, the reaction was stopped and samples were analysed by SDS-PAGE followed by autoradiography (top panels) or Coomassie staining (bottom panels). In the right panel, BSA was added so that the total of MCM-BP plus BSA was 4 µg. Note that BSA co-migrates with MCM-BP in SDS-PAGE. Phosphorylation reactions were also performed with 4.0 µg MCM-BP alone (lane 2, left panel) and 0.5 µg MCM2 alone (lane 1, right panel). Control reactions lacking DDK were also performed to show that labeling was due to DDK activity (lanes 1 and 3, left panel and lane 2, right panel). B. Reactions were performed as in A except that 0.5 µg MCM2 was used in place of MCM4,6,7. C. Quantification of ^32^P-labelled bands corresponding to MCM2 (from B) or MCM4 and 6 (from A) or MCM7 (from A) are shown where #1 and #2 indicates the results from A left panel and right panel, respectively. Values are shown relative to labeling in the absence of MCM-BP (set as 100%). D. 2 µg of MCM4,6,7 or MCM2-7 complex was incubated with or without 2 µg of MCM-BP, then kinase assays were performed with 250 ng of DDK or no DDK as in A. E. ^32^P in the MCM2 or MCM3-7 bands in D was quantified and background labeling from reactions without DDK was subtracted from the same reactions with DDK. The percentage of DDK-specific ^32^P incorporation in the presence of MCM-BP is shown relative to that in the absence of MCM-BP.

Most of the MCM proteins that interact with Dbf4 are substrates for DDK. Therefore we tested whether MCM-BP was phosphorylated *in vitro* by purified DDK, generated by co-expressing Dbf4 and Cdc7 in *E.coli* [31] and isolating the complex as described in Materials and Methods. After incubation with this kinase in the presence of γ-^32^P-ATP, no labelling of MCM-BP was detected (Figure 7A, left panel, lane 2), although the purified MCM4,6,7 complex (left panel, lane 4) and purified MCM2 (Figure 7A, right panel, lane 1 and Figure 7B, lane 2) were clearly phosphorylated by the same kinase preparation. In addition, no labelling of MCM4,6,7 (Figure 7A, left panel, lane 3) or MCM2 (Figure 7B, lane 1) was observed when DDK was left out of the reaction, showing that the labelling was not due to a contaminating kinase in the MCM preparations. Therefore the results indicate that MCM-BP is not a substrate for DDK.

We next asked whether MCM-BP might interfere with MCM phosphorylation by DDK through its interactions with Dbf4 or MCM proteins or both. To this end we preincubated increasing amounts of MCM-BP with a constant amount of MCM4,6,7 (Figure 7A, left panel, lanes 5–8) or MCM2 (Figure 7B, lanes 3–6) then performed the DDK kinase reaction as above. We found that MCM-BP caused a titratable decrease in phosphorylation of MCM4,6 and 7 but had only a subtle effect on phosphorylation of MCM2. To account for any nonspecific effects of increasing amounts of protein in the reactions, we repeated the MCM-BP titration with MCM4,6,7 but balanced the amount of MCM-BP added with BSA to maintain a constant amount of protein in each reaction (Figure 7A, right panel, lanes 4–7). When the DDK kinase assays were performed on these samples, the results were the same as for the initial MCM-BP titration. ^32^P-containing bands corresponding to MCM4 and 6 (which migrate too close together to distinguish), MCM7 and MCM2 from each experiment with MCM-BP titration were quantified and are shown in the graph in Figure 7C. The results show that MCM-BP can limit the phosphorylation of MCM4,6 and 7 by DDK but that MCM2 phosphorylation is not significantly affected.

Since the MCM complex that functions in DNA replication contains all six MCM subunits, we also performed the DDK *in vitro* phosphorylation assays with MCM2-7 hexamers, generated by insect cell co-expression and purification as in Sakwe et al [21]. DDK phosphorylation of proteins in the MCM2-7 complex was examined in the presence and absence of MCM-BP and compared to that of MCM4,6,7 (Figure 7D). Control reactions lacking added DDK, showed that there was a small amount of MCM phosphorylation activity in the MCM2-7 preparation (lanes 5 and 7), however the addition of DDK greatly increased the phosphorylation of the MCM proteins (compare lanes 5 and 6). As seen for the MCM4,6,7 subcomplex, the presence of MCM-BP greatly decreased the phosphorylation of MCM proteins migrating in the region of MCM3-7 (we cannot distinguish which band is labelled due to the close migration of these proteins; compare lanes 6 and 8). However, phosphorylation of the MCM2 band in the MCM2-7 complex was much less affected by MCM-BP (compare lanes 6 and 8). Quantification of the ^32^P in these bands followed by subtraction of background labelling, showed that MCM-BP decreased DDK phosphorylation of MCM3-7 proteins by 73% and MCM2 by 17% in the context of the MCM2-7 complex (Figure 7E).

## Discussion

MCM-BP is known to make important contributions to DNA replication presumably through its interaction with MCM complexes, although the nature of these interactions have not been well defined. Here we have shown that MCM-BP has some propensity to interact with any of the MCM 2 to 7 proteins, although it appears to interact most stably with MCM4 and MCM7. These interactions can lead to the dissociation of MCM2-7 hexamers *in vitro* and can affect the solubility or behaviour of some individual MCM proteins. We also identified an interaction with the Dbf4 regulatory component of the DDK kinase, known to phosphorylate MCM proteins and to play multiple roles in DNA replication.

Assessment of the physical interactions of MCM-BP with MCM proteins expressed in insect cells indicated that, while MCM-BP can interact with any individual MCM protein, it interacts most strongly with MCM4 and MCM7. A preferential interaction with MCM7, but not MCM4, was previously reported for *Xenopus* MCM-BP. Sucrose gradient analysis of *Xenopus* egg extracts identified a complex containing MCM-BP and MCM7, but not other MCMs, suggesting preferential association of MCM-BP with MCM7 [22]. Furthermore, the interaction between MCM-BP and MCM7 was shown in GST pulldown assays to involve the MCM box of MCM7 [22]. Since the MCM box is conserved in all MCM proteins, this finding fits with our observations that MCM-BP has some capacity to interact with all MCM proteins. The interaction between *Xenopus* MCM-BP and MCM7 was also found to be stable up to 0.8 M NaCl, consistent with our findings that MCM-BP interactions with MCM proteins are very salt stable. This is typical of interactions between MCM proteins which remain intact in up to 1 M NaCl [5], [32], [33].

By assaying pairwise MCM protein interactions and determining those that result in ATPase activity, the order of the MCM proteins in the hexameric ring has been determined [12], [34]. Further work has suggested that a ‘gate’ exists between MCM2 and MCM5 whose state dictates the open and closed conformations of the MCM complex [35], [36]. A ‘closed’ MCM 2/5 gate has been shown to promote the helicase activity of the MCM complex by activating the MCM4/7 motor, located 180 degrees across from the MCM2/5 gate. Our finding that MCM-BP binds most prominently to MCM4 and MCM7 suggests that MCM-BP may contact the hexameric ring through these proteins across from the gate, and further regulate helicase activity. Considerable evidence indicates that MCM4 and MCM7 are key proteins in the MCM complex and important targets for MCM functional regulation. Phosphorylation of MCM4 serves as a means of regulating the MCM complex, as MCM4 phosphorylation by cyclin A/Cdk2 inactivates the helicase activity [37], [38]. MCM7 is also of particular importance for the activity of the MCM2-7 hexamer as it contributes to two ATPase active sites (with MCM4 and MCM3) [12], [35]. In addition, protein interactions with MCM7 are known to regulate the activity of the MCM complex. For example, an interaction between cyclin A and MCM7 promotes S-phase entry [39], while DNA replication is inhibited by binding of the retinoblastoma protein to MCM7 [40]. MCM7 also appears to have functions independent of the MCM complex, as MCM7 (but not other MCM proteins) is important for Chk1 signalling through its interactions with Rad17 and ATR-interacting protein (ATRIP) [19], [41]. In addition, a role for MCM7 in hypoxia was recently identified in which MCM7 binds and induces the degradation of hypoxia-inducible factor 1 (HIF-1) [20]. Therefore, in addition to dissociating the MCM complex, MCM-BP interactions with MCM7 and MCM4 may regulate the functions of the MCM complex as well as other roles of these proteins.

Our finding that MCM-BP can affect the solubility of some individual MCM proteins raises the possibility that MCM-BP could serve a chaperone-like function affecting the pool of MCM proteins that are not assembled on chromatin. This property of MCM-BP might also be important for promoting the dissociation of MCM complexes. Previous studies on the *Xenopus* and *S. pombe* versions of MCM-BP supported a role for MCM-BP in dissociating MCM hexamers [22], [23]. We have now shown that this property is also intrinsic to human MCM-BP and that MCM complexes from either G1 (the dominant phase in log-phase cells) or various stages of S-phase can be disrupted by MCM-BP.

The co-purification of MCMs3-7 with MCM-BP from a variety of organisms suggests that MCM-BP can form a hexameric complex with these MCM proteins [21], [22], [23], [24]. In addition, MCM-BP can form a complex with the MCM4,6,7 core helicase when co-expressed with them in insect cells [21]. However, examination of the state of MCM-BP complexes by glycerol or sucrose gradient sedimentation of cell extracts has given variable results in different organisms. Analysis of *Xenopus* interphase egg extracts detected a complex of MCM-BP and MCM7 but did not detect MCM-BP in large MCM complexes [22]. Li et al [24] found that *pombe* Mcb1 co-migrated with MCM4 and MCM6 but the migration of other MCM proteins was not examined. Our analysis of human cell extracts indicated that most of the MCM-BP did not co-migrate with the MCM hexamers, nor did we detect obvious complexes between MCM-BP and single MCM subunits. However, a small proportion of the MCM-BP appeared as a distinct high-molecular weight peak in mid to late S phase, that also contained the MCM proteins. This observation may be relevant for MCM complex unloading since previous data suggests that MCM-BP promotes the dissociation of MCM complexes from the chromatin in mid to late S [22]. Our results suggest that, in human cells, higher order complexes between MCM-BP and MCM proteins are not constitutive but may form transiently during S phase.

Interactions between MCM proteins and DDK are known to be important for origin activation. Since MCM-BP forms complexes with MCM proteins and can be detected on cellular origins at G1/S (when DDK is active) [21], we wanted to examine whether MCM-BP interacted with the Dbf4 regulatory component of this kinase. We found that MCM-BP interacted with Dbf4 in yeast 2-hybrid assays and upon co-expression in insect cells, and that the two endogenous proteins co-immunoprecipitated from human cells. While the interaction of DDK with the MCM complex is known to result in the phosphorylation of MCM2, MCM4 and MCM6, an important event in origin activation [6], [7], [8], [9], [10], [42], [43], we found that MCM-BP was not phosphorylated by DDK *in vitro* whereas MCM2, 4, 6 and 7 were phosphorylated under the same conditions. While MCM7 phosphorylation by DDK has not been widely studied, budding yeast MCM7 has also been reported to be a substrate for DDK [44]. Interestingly, we found that MCM-BP inhibited DDK phosphorylation of the MCM4,6,7 complex in a dose-dependent manner, suggesting that the interaction of MCM-BP with DDK and/or the MCM4,6,7 complex interfered with phosphorylation. Indeed we have previously shown that MCM-BP forms a complex with MCM4,6,7 that is stable to glycerol gradient sedimentation [21]. The finding that DDK phosphorylation of MCM2 was less affected by MCM-BP suggests that the strong interaction of MCM-BP with the MCM4,6,7 complex is at least partly responsible for this inhibition, as opposed to having a direct effect on cdc7 activity. We also confirmed these findings in MCM2-7 hexamers, where MCM-BP had little effect on MCM2 but inhibited DDK phosphorylation of one or more of the other MCM proteins. The ability of MCM-BP to affect DDK phosphorylation of MCM 4 and 6 may be relevant for origin activation where these phosphorylation events have been shown to be important. MCM-BP was found to be preferentially associated with the lamin B2 origin at G1/S where it could conceivably influence DDK phosphorylation at this stage of the cell cycle [21]. In addition, DDK has been found to be important for S-phase progression through MCM4 phosphorylation, Chk1 checkpoint signalling and replication fork restart after a prolonged S-phase checkpoint, raising the possibility that MCM-BP might also impact these processes through regulation of DDK phosphorylation [29], [45], [46], [47], [48], [49], [50]. Future studies on the role of the MCM-BP/Dbf4 interaction will be important for elucidating the mechanism of action of MCM-BP and its functions in DNA replication.

## Materials and Methods

### Antibodies

Antibodies used in this report were MCM2 (Santa Cruz 9839), MCM3 (Santa Cruz 9850), MCM4 (Santa Cruz 22779), MCM5 (Santa Cruz 165993), MCM6 (Santa Cruz 9843), MCM7 (Santa Cruz, 22782), Dbf4 (Santa Cruz 11354) and Flag M2 (Sigma). The antibody against MCM-BP has been described previously [21]. For Western blots, all secondary antibodies were coupled to horseradish peroxidase (obtained from Santa Cruz Biotechnology) and detected by ECL.

### Baculoviruses

Baculoviruses expressing individual MCM proteins or MCM-BP with N-terminal 6-histidine tags (MCM-BP, MCM3) or with 6-His tags in combination with a triple FLAG tag (MCM5 and MCM7) or a StrepII tag (MCM4) or a HA tag (MCM6) were generated using the pfastBacHT system as described in Sakwe et al [21]. Baculoviruses expressing MCM-BP with no tags or an N-terminal StrepII-tag or Dbf4 with 6-His and FLAG tags were also generated using this system. MCM-BP with no tags was generated from pfastBacHT.MCM-BP by digesting this plasmid with Rsr II and Nco I (which excises the 6-His tag), filling in the ends with DNA polymerase I Klenow fragment (NEB) and religating. StrepII-MCM-BP was generated by inserting a synthetic cassette containing the StrepII tag between the Rsr II and Nco I sites of pfastBacHT. MCM-BP. A baculovirus expressing Dbf4 with an N-terminal 6-His tag followed by a triple FLAG tag was generated using the pfastBacHT system as described for MCM7 and MCM5 in Sakwe et al [21].

### Assay of MCM-BP Interactions with Individual MCM or Dbf4 Proteins in Insect Cells

High Five insect cells were coinfected with baculoviruses expressing MCM-BP and individual affinity-tagged MCM or Dbf4 proteins. Where indicated, the tagged MCM proteins were also expressed on their own (using twice as much baculovirus as in co-infections to keep the total amount of baculovirus constant). In Figure 3C, hexahistidine-tagged MCM-BP was co-expressed with untagged MCM proteins. After 3 days, cells were harvested, washed twice with PBS and lysed in 50–100 mM Tris-HCl, pH 8.0, 150 mM NaCl, 5% glycerol, 0.5% TritonX-100, complete protease inhibitor mixture (P8340 from Sigma), and 2 mM EDTA (omitted in experiments using Ni-NTA resin). The lysates were clarified by centrifugation at 13,000×g for 15 minutes then the soluble fractions were incubated with Ni-NTA (Qiagen), anti-FLAG M2 (Sigma), anti-HA (Santa Cruz) or StrepT-actin (Qiagen) resin (depending on the tag on the protein of interest) for 1 hr at 4°C with mixing. In Figure 3A–C, the NaCl concentration of the clarified lysate was increased as indicated or deoxycholate was added to 0.1 or 0.5% prior to incubation with the resin. The resin was then washed three times for 2 minutes each with 40 volumes of Buffer A (50 mM Tris-HCl, pH 8.0, 300 mM NaCl and 5% glycerol) and eluted with Buffer A containing 250 mM imidazole (for Ni-NTA), 0.5 mg/ml 3-FLAG peptide (for anti-FLAG M2; Sigma), 1% SDS (for anti-HA) or 5 mM destiobiotin (for StrepT-actin). In Figure 3D, hexahistidine-tagged MCM4 or MCM7 was co-expressed with untagged MCM-BP then cells were lysed and incubated with nickel resin as above (150 mM NaCl condition). The resin was then washed with Buffer A then incubated with 3 volumes Buffer A containing either 0, 0.1% or 0.2% CHAPS (Sigma) for 30 min at room temperature. The eluted proteins were then analyzed by SDS-PAGE and Coomassie staining, Western blotting or glycerol gradient centrifugation.

### Glycerol Gradient Analysis of MCM-BP-MCM Dimer Pairs

Dimer pairs of MCM-BP with an MCM protein were isolated as described above and 150–200 µg was applied to a 12 ml 15–35% glycerol gradient in 20 mM Tris-HCl, pH 7.5, 100 mM NaCl, 0.1 mM EDTA, 0.1 mM PMSF, and 2 mM DTT. After centrifugation at 34, 000 rpm in a SW41 rotor (Beckman) for 16 hours at 4°C, 24 500 µl fractions were collected from the top of the gradient and 35 µl of each fraction was analyzed by SDS-PAGE and silver staining.

### Glycerol Gradient Analysis of MCM Proteins and MCM-BP in HeLa Cell Lysates

HeLa cells (purchased from ATCC) were either collected in log phase or were synchronized at G1/S by double thymidine block. To synchronize the cells, thymidine (Sigma) was added to final concentration of 2 mM for 19 hours, followed by two washes in PBS and release into complete DMEM for 10 hours. Thymidine was then added again to 2 mM for 17 hours and cells were released into complete medium for 2, 4 or 6 hours. HeLa cells were collected by scraping, washed twice with PBS and lysed in five volumes of 50 mM Tris-HCl pH 8.0, 150 mM NaCl, 0.1% Triton X-100 (v/v), 2 mM EDTA pH 8.0, 5% glycerol (v/v) and complete protease inhibitor (Sigma p8340). The lysate was sonicated for 5 seconds at 50% amplitude and clarified by centrifugation at 16,000×g for 10 minutes. Two mg of lysate was then loaded onto a 12 ml 15–35% glycerol gradient in 25 mM Tris-HCl pH 7.5, 100 mM NaCl, 0.1 mM EDTA pH 8.0, and 2 mM DTT. Where indicated, 30 µg of His-MCM-BP purified from insect cells as previously described [21] was added to 2 mg of lysate and incubated for 30 min at 4°C prior to loading on the gradient. Glycerol gradients were subjected to centrifugation at 34,000 rpm in a Beckman SW-41 rotor for 23 hours at 4°C, then 24 500 µl fractions were collected from the top of the gradient. A 35 µl sample of each fraction was analyzed by SDS-PAGE and Western blotting. DNA content analysis was also performed on samples of the cells to verify cell cycle stages. To this end, cells were fixed in 70% ethanol overnight at −20°C, washed with PBS with 0.5% BSA, treated with 100 µg/ml RNase A for 1 hour at 37°C and stained with 50 µg/ml propidium iodide. Samples were analyzed at the University of Toronto, Faculty of Medicine Flow Cytometry Facility, using a FACS Calibur flow cytometer (BD Biosciences) and data was collected using CellQuest software. Cell cycle analysis was performed using FlowJo software (Treestar Inc.). For the 6 hour time point, MCM-BP was immunoprecipitated overnight with anti-MCM-BP rabbit serum from 200 µl of fraction 14 and 15 diluted with 400 µl of Buffer B (50 mM Tris, pH 8.0, 150 mM NaCl). Following by a 2 hour- incubation with protein A/G plus beads (Santa Cruz 2003) and three washes with Buffer B, bound proteins were eluted in SDS sample buffer and analyzed by Western blotting.

### Purification of MCM2, MCM4,6,7, MCM2-7 and MCM-BP for DDK Assays

MCM2 and the MCM4,6,7 and MCM2-7 complexes were purified from High Five insect cells as previously described [21]. MCM-BP was expressed in *E.coli* from pET15b in which the MCM-BP cDNA was inserted between the NdeI and BamHI restriction sites. BL21DE3 cells [51] were transformed with pET15b.MCM-BP and expression of MCM-BP was induced for 18 hrs at 15°C by the addition of 1 mM IPTG. The bacteria were lysed in 25 mM HEPES, pH 7.8, 500 mM NaCl, 5 mM imidazole, 10 mM mercaptoethanol, 0.2% TritonX-100, 0.2% CHAPS, 5% glycerol, and complete protease inhibitor mixture (Sigma P8340) and clarified by centrifugation in a Sorvall SS-34 rotor at 13000 rpm for 30 minute. The clarified lysate was loaded onto a Ni-NTA column (Qiagen), washed with 10 mM imidazole, and eluted with 250 mM imidazole in lysis buffer. The His-tag was then removed from MCM-BP by adding thrombin (1% w/w) and dialysing overnight against 25 mM HEPES, pH 7.5, 150 mM NaCl, 2.5 mM CaCl_2_, 5% glycerol, 10 mM mercaptoethanol. MCM-BP lacking the His-tag was separated from the His tag by flowing through a second Ni-NTA column. The MCM-BP was then applied to a Superdex 200 gel filtration column in 20 mM HEPES pH 7.8, 200 mM NaCl, 5 mM DTT. Peak fractions were collected and concentrated with an Amicon Ultra centrifugal filter devices (Millipore).

### DDK Purification

pET-30a expressing both Myc-Cdc7 and 6His-Dbf4 was a gift from Drs. Jerard Hurwitz and Joon-Kye Lee and was previously described [31]. This plasmid was used to transform BL21(DE3) CodonPlus – RIL cells (Novagen) and protein expression was induced by IPTG addition for 18 hours at 15°C. The cells were lysed in 50 mM Tris-HCl, pH 8.0, 200 mM NaCl, 5% glycerol, 10 mM imidazole and complete protease inhibitor mixture and clarified by centrifugation in a Sorvall SS-34 rotor at 13000 rpm for 30 minute. The clarified lysate was loaded onto a Ni-NTA column (Qiagen), washed with the lysis buffer, eluted with lysis buffer containing 250 mM imidazole and dialyzed overnight against 25 mM Tris-HCl, pH 8.0, 150 mM NaCl, 5% glycerol, 1 mM DTT and 0.1 mM EDTA.

### DDK in vitro Phosphorylation Assays

Purified MCM4,6,7 complex (2 µg), MCM2-7 complex (2 µg) or MCM2 (0.5 µg) was incubated with 0.5, 1.0, 2.0 or 4.0 µg purified MCM-BP or no MCM-BP for 15 minutes on ice in a 8 µl volume, then combined with 250 ng purified DDK in a 20 µl final volume containing 25 mM HEPES, pH 7.5, 10 mM magnesium acetate, 0.1 mg/ml BSA, 1 mM DTT, 50 mM NaCl, 50 µM ATP, 0.1 µl of 3000 ci/mmol [γ-32-P]ATP (NEG 502A PerkinElmer) and complete phosphatase inhibitor cocktail (Thermo Scientific). After 30 min at 30°C, the reaction was stopped by heating at 95°C for 3 minutes in SDS loading buffer. Proteins were separated by 8% SDS-PAGE. ^32^P-labelled bands were imaged with a Typhoon Laser Scanner (GE HealthCare life Sciences) and quantified using ImageQuant. The positions of individual proteins were visualized by Coomassie staining.

### Yeast 2-hybrid Assays

MCM-BP was PCR amplified from pMZI.MCMBP [21] and this cDNA was used to replace the EBNA1 cDNA in pLexA-EBNA1 (described in [52]). Specifically, EBNA1 cDNA was excised from pLexA-EBNA1 with Nde I and Bgl II and the MCM-BP cDNA was inserted between these sites. MCM4 was PCR amplified from MCM4 in pOTB7 (purchased from ResGen; Invitrogen) and cloned between the Nco I and Bam HI sites of pACTII. MCM7 was amplified from pMZI.MCM7 [21] and inserted in the Xma I site of pACTII. Dbf4 in pBluescriptR and Plk1 in pOTB1 were obtained from The Centre for Applied Genomics (TCAG), The Hospital for Sick Children, Toronto and used to PCR amplify the cDNA. The amplified Dbf4 or Plk1 cDNAs were inserted between the Sfi I and Eco RI sites of pACTII. Two-hybrid assays were performed as described in Nayyar et al [53]. Briefly, *S. cerevisiae* strain L40a (*MAT*a *trp1 leu2 his3 LYS2::lexA-HIS3 URA3::lexA-lacZ*) [54] was transformed with pLexA.MCMBP and with pACTII expressing the indicated protein (fused to the GAL4 activation domain) or with empty pACTII (negative control). Yeast were grown overnight in medium selective for both plasmids (lacking Trp and Leu) and 10-fold serial dilutions of the cultures were spotted and grown on plates lacking Trp and Leu or lacking Trp, Leu and His and containing 2 mM aminotriazole (to select for yeast expressing histidine and reduce background levels of histidine).

### Immunoprecipitations from Human Cells

4×10^6^ HeLa cells were harvested by trypsinization and lysed in 50 mM Tris HCl pH 8, 150 mM NaCl, 5% glycerol, 5 mM EDTA and 0.1% Triton X-100 for 30 minutes on ice. The lysate was clarified by centrifugation at 15,000 rpm for 10 minutes. 1 mg of lysate was incubated overnight at 4°C with either MCM-BP antibody or control IgG (Santa Cruz 2345). Subsequently, 30 µl Protein A/G agarose beads (Santa Cruz 2003) was added and incubated for 2 hours at 4°C, then the beads were washed three times in the lysis buffer. Immunoprecipitated proteins were eluted from the beads in SDS sample buffer and analysed by Western blotted using antibodies against MCM2, MCM4, MCM-BP and Dbf4.
